# Inadvertent Occlusion of the Anterior Choroidal Artery Explains Infarct Variability in the Middle Cerebral Artery Thread Occlusion Stroke Model

**DOI:** 10.1371/journal.pone.0075779

**Published:** 2013-09-17

**Authors:** Damian D. McLeod, Daniel J. Beard, Mark W. Parsons, Christopher R. Levi, Mike B. Calford, Neil J. Spratt

**Affiliations:** 1 University of Newcastle and Hunter Medical Research Institute, Callaghan, New South Wales, Australia; 2 Hunter New England Local Health District, New Lambton Heights, New South Wales, Australia; National University of Singapore, Singapore

## Abstract

Intraluminal occlusion of the middle cerebral artery (MCAo) in rodents is perhaps the most widely used model of stroke, however variability of infarct volume and the ramifications of this on sample sizes remains a problem, particularly for preclinical testing of potential therapeutics. Our data and that of others, has shown a dichotomous distribution of infarct volumes for which there had previously been no clear explanation. When studying perfusion computed tomography cerebral blood volume (CBV) maps obtained during intraluminal MCAo in rats, we observed inadvertent occlusion of the anterior choroidal artery (AChAo) in a subset of animals. We hypothesized that the combined occlusion of the MCA and AChA may be a predictor of larger infarct volume following stroke. Thus, we aimed to determine the correlation between AChAo and final infarct volume in rats with either temporary or permanent MCA occlusion (1 h, 2 h, or permanent MCAo). Outbred Wistar rats (n = 28) were imaged prior to and immediately following temporary or permanent middle cerebral artery occlusion. Presence of AChAo on CBV maps was shown to be a strong independent predictor of 24 h infarct volume (β = 0.732, *p* <0.001). This provides an explanation for the previously observed dichotomous distribution of infarct volumes. Interestingly, cortical infarct volumes were also larger in rats with AChAo, although the artery does not supply cortex. This suggests an important role for perfusion of the MCA territory beyond the proximal occlusion through AChA-MCA anastomotic collateral vessels in animals with a patent AChAo. Identification of combined MCAo and AChAo will allow other investigators to tailor their stroke model to reduce variability in infarct volumes, improve statistical power and reduce sample sizes in preclinical stroke research.

## Introduction

Studies in both human and animals have reported large variability in the vascular anatomy of the brain. Of significance in the field of ischemic stroke research is the anatomy of the middle cerebral artery (MCA) and adjacent branches, in particular the anterior choroidal artery (AChA). The AChA supplies blood to the globus pallidus, the internal capsule, choroid plexus and has anastomotic connections with the MCA in rats and humans [1], [2]. Despite the AChA territory being well defined, there is substantial variability in the origin of this vessel. The AChA predominantly originates from the internal carotid artery (ICA) in close proximity to the MCA in rats and humans [1]–[3].

Intraluminal thread occlusion of the MCA in the rat is the most common experimental model of stroke. This technique is performed by inserting an occluding monofilament suture via the ICA to occlude the origin of the MCA [4]. Advantages of this technique include lack of craniotomy and control over occlusion and reperfusion. One disadvantage of this model is the large variability in infarct volume. Factors thought to influence infarct volume variability include genetics [5], blood pressure [6], collateral blood flow [7], duration of vessel occlusion, and type of intraluminal thread used [8].

A new observation was made while developing our perfusion computed tomography (CTP) rat stroke model (intraluminal MCAo) [9], [10]. In a subset of animals, there was loss of arterial-intensity signal in the distribution of the anterior choroidal artery (AChA) on cerebral blood volume (CBV) maps, in addition to the expected loss of signal in the MCA territory. We hypothesized that the combined occlusion of the MCA and AChA may be a predictor of larger infarct volume at 24 h following stroke. Thus we aimed to determine the correlation between AChAo and final infarct volume, in rats with either temporary or permanent MCA occlusion (1 h, 2 h, and permanent MCAo). The aims of the project were successfully achieved.

## Methods

### Ethics Statement

All surgical and experimental protocols were in accordance with the Australian Code of Practice for the Care and Use of Animals for Scientific Purposes and were approved by the University of Newcastle (UoN) Animal Care and Ethics Committee.

### Surgical Procedures

Male outbred Wistar rats (300–500 g) (Animal Services Unit, UoN) underwent MCAo, using the silicone-tipped intraluminal thread occlusion method [11]. Each 4-0 monofilament occluding thread had a 3 mm silicone tip (0.35 mm diameter). Animals were anesthetized with isoflurane (5% induction, 1.5 – 2.5% maintenance during surgery and 1.0–1.5% during imaging) and a 50:50 mix of nitrous oxide and oxygen via facemask. Animals were subject to either permanent MCAo (n = 13), or 1 hr (n = 8) or 2 hr (n = 7) temporary MCA occlusion, with reperfusion achieved by gentle withdrawal of the occluding thread. Details of thread insertion for MCAo on the CT scanning table have been previously reported [9]. All animals had a jugular venous catheter inserted for injection of radio-opaque contrast during imaging [9].

### CT Image Acquisition, Processing and Analysis

Detailed methods and perfusion computed tomography (CTP) data have been reported for this cohort of animals [9], [10]. All imaging was performed using a Siemens 64 slice CT scanner (Siemens, Erlangen, Germany) with a 512×512 matrix, 50 mm field of view (FOV) with twelve 2.4 mm slices.

**Figure 1:**
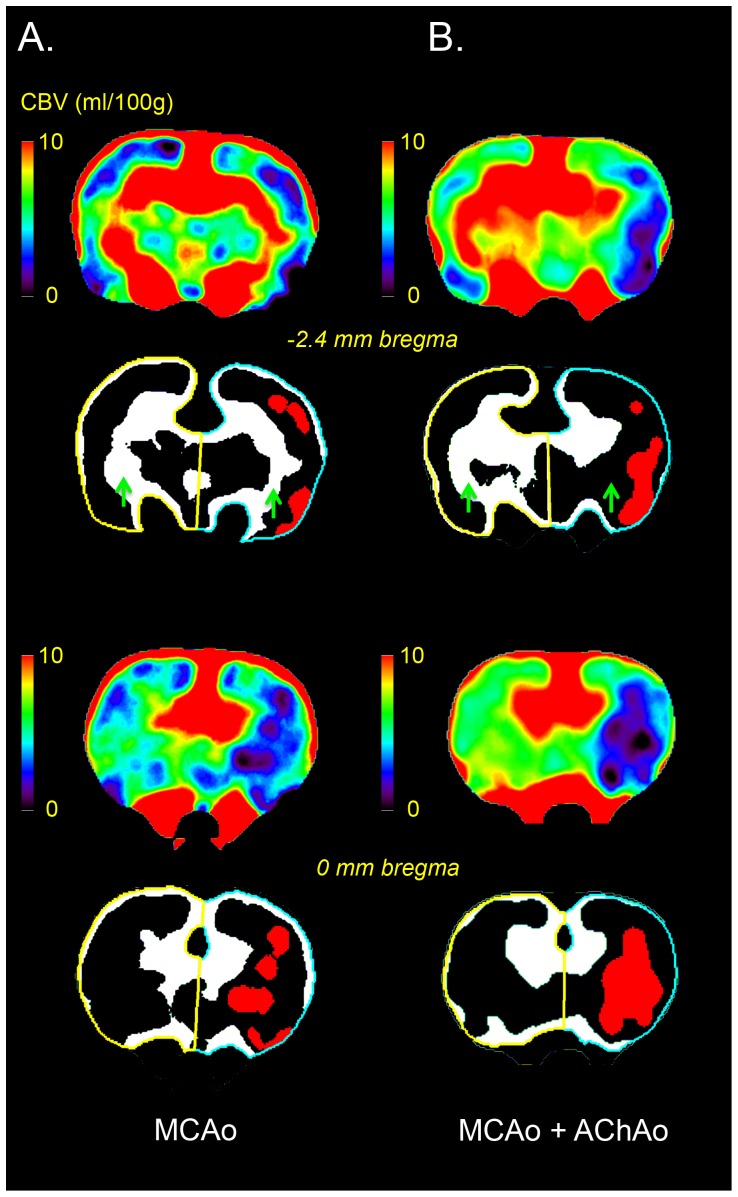
Perfusion Computed Tomography (CTP) analysis method. Cerebral blood volume (CBV) maps (coronal plane) from two rats taken immediately after intraluminal thread occlusion of the middle cerebral artery (MCAo). The animal in column A is seen to have MCAo with preserved AChA blood volume whereas the animal shown in column B has both MCAo and anterior choroidal artery occlusion (AChAo). For each rat, 2 bregma levels are shown, with the top 2 images showing the more caudal sections. At each level the colour coded CBV maps are shown on top, and underneath a CBV threshold of 10 ml/100 g was applied to all CBV maps to remove large blood vessels, and ROIs encompassing the left (contralateral, yellow outline) and right (ipsilateral, blue outline) hemispheres were generated. Voxels below the mean contralateral CBV were thresholded out to leave the voxels with higher CBV (white). Green arrows indicate the location of the AChA territory. Lack of CBV voxels in the ipsilateral AChA territory (column B, row 2) indicates occlusion of the AChA. For illustrative purposes, a CBV threshold of 2 ml/100 g [19] has been applied to the CBV maps to indicate the areas predicted to infarct within the MCA territory (red).

Post-processing of perfusion data was performed using MIStar imaging software (Apollo Medical Imaging Technology, Melbourne, Australia), as previously described [9]. In brief, cerebral blood flow (CBF), cerebral blood volume (CBV), and mean transit time (MTT) maps were generated from CTP images recorded within 5 minutes after MCAo. The two CTP slices that were analyzed were located at 0 mm bregma, and at 2.4 mm caudal to bregma. This allowed assessment of perfusion changes in the MCA and AChA territories. First, to exclude voxels containing large blood vessels the standard CBV large vessel threshold (10 ml/100 g) [9], [10] was applied. This threshold did not exclude voxels within the AChA territory, however this territory was clearly visible on CBV colour maps. Therefore, a more stringent threshold was used to provide an objective marker of presence or absence of AChA occlusion. To do this, regions-of-interest (ROIs) encompassing the left (contralateral) and right (ipsilateral) hemispheres were generated (Figure 1). To determine the presence of concurrent AChAo, a threshold greater than or equal to the mean contralateral CBV was applied. Lack of signal in the ipsilateral AChA territory on these maps indicated occlusion of the AChA. Data analysis was performed by an observer (DDM) blinded to infarct volume outcome.

### Histology

At 24 hours following MCA occlusion, rats were euthanased by transcardial perfusion fixation with 4% paraformaldehyde. This was followed by histological processing, hemotoxylin-eosin (H&E) staining, scanning at 20x objective on a digital slide scanner (Aperio Technologies Inc., Vista, CA, USA), and infarct quantification, as previously described [9]. Infarct volumes were corrected for edema using the formula: corrected infarct vol. (mm^3^)  =  infarct vol. x (contralateral vol./ipsilateral vol.). Infarct probability maps were generated for the pMCAo group at two bregma levels (–0.3 mm and –2.3 mm bregma) using Adobe Photoshop Version 13.0 (Adobe Systems Incorporated). The Photoshop Warp tool was used to transform the histological outlines to fit the stereotaxic atlas template (Paxinos and Watson [12]). The traced infarct outlines were filled with the same grey colour and the opacity was set to 24%. Sections from subsequent animals were overlaid.

### Statistics

Statistical tests were performed with Graphpad Prism 6 (CA, USA) and SPSS 21.0 (IBM, USA). Due to the retrospective nature of the study, D’Agostino & Pearson omnibus normality tests were performed on all data prior to further statistical analyses. If data was normally distributed (alpha > 0.05), two-tailed *t-*tests were performed. The effect of MCAo vs. MCAo + AChAo on infarct volume within each of the 1 h, 2 h, and pMCAo groups, and in all animals combined were analyzed. All other statistical analyses between groups were done with two-tailed *t-*tests.

Regression analysis was used to determine independent predictors of infarct volume. The predictors of interest were MCA occlusion duration, the patency of AChA (i.e. occluded or not occluded), and weight. Each of these predictors was examined with a Pearson correlation analysis, and those with *p*<0.10 were included in a multiple linear regression model. The standardized coefficients (β-value) and *p*-values are presented to indicate the significant predictors of infarct volume. Statistical significance was accepted at *p*<0.05. Data is presented as mean ± SD.

## Results

**Figure 2:**
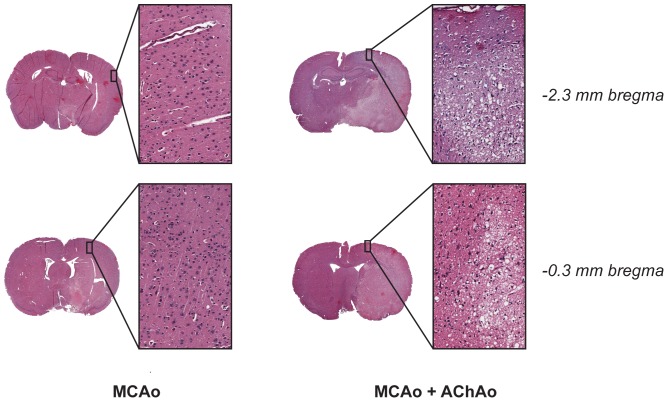
Histology sections from two pMCAo rats 24 h after stroke. Hemotoxylin-eosin stained coronal sections scanned at 20x objective. The animal with the MCAo + AChAo (right column) had prominent subcortical and cortical infarction (pyknotic, dark, shrunken nuclei: expanded regions-of-interest); the animal with MCAo alone (left column) had subcortical infarction, but no infarction within similar cortical locations.

Successful occlusion of the MCA was observed in all animals (n = 28) by visual inspection of the CTP maps (Figure 1). Infarction was confirmed in all animals with H&E histology at 24 h (Figure 2). A subset of animals (n = 10) had no CBV above the mean contralateral CBV in the ipsilateral AChA territory, indicating AChAo (Figure 1B).

**Figure 3:**
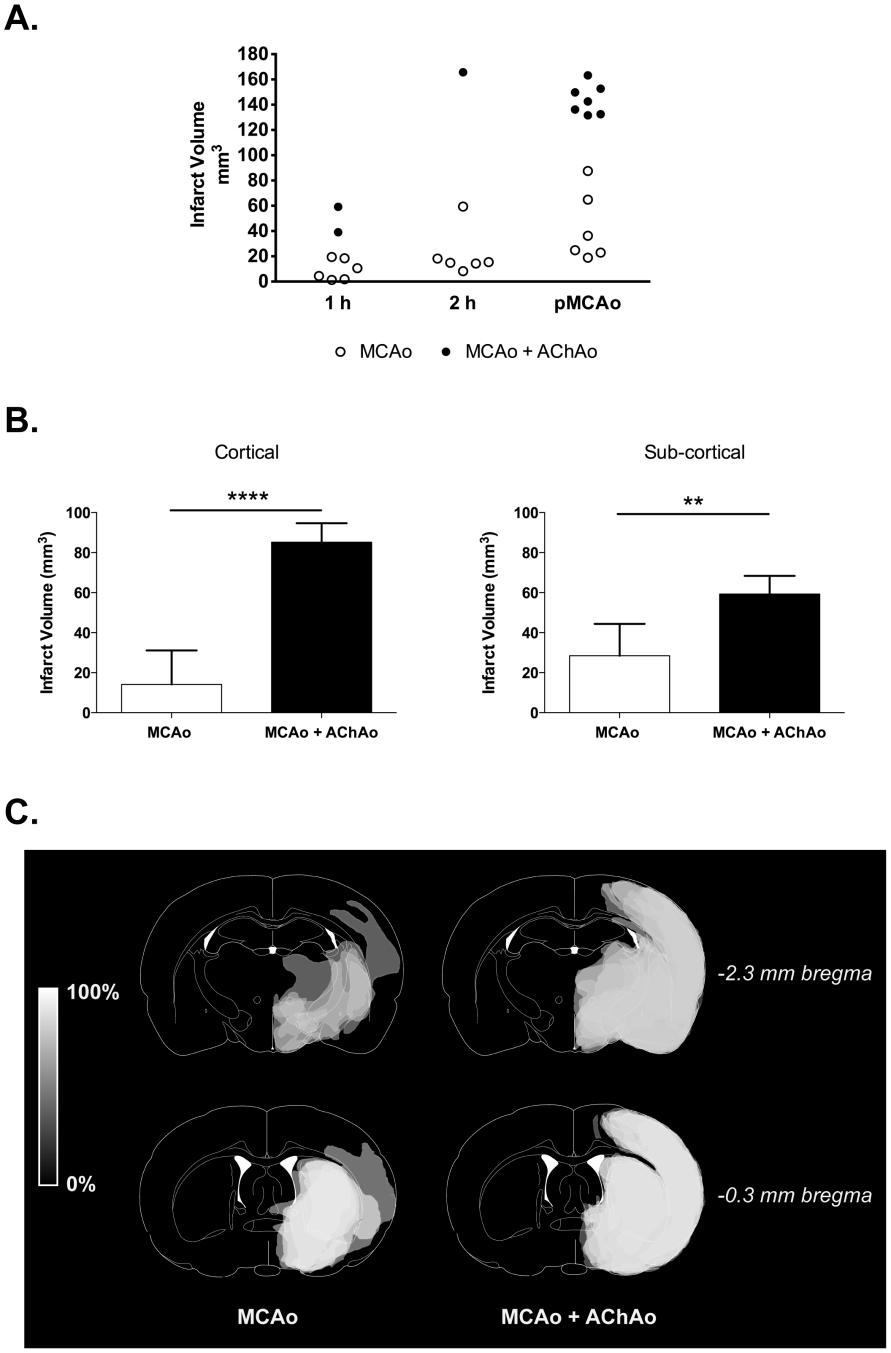
Effect of concurrent anterior choroidal artery occlusion on infarct volume after temporary or permanent MCA occlusion. Outbred Wistar rats underwent 1 h (n = 8), 2 h (n = 7) or permanent MCAo (n = 13) of the right middle cerebral artery (MCAo) using a silicone-tipped intraluminal filament. A. Infarct volume was quantified with histology at 24 h. Presence of anterior choroidal artery occlusion (AChAo) was determined by cerebral blood volume (CBV) map analysis. B. Cortical and sub-cortical infarct volume distribution in pMCAo animals (mean + sd). C. The traced infarct (seen in grey) from animals in the pMCAo group were overlaid so that lighter regions represent areas more commonly infarcted.

Infarct volumes from the pMCAo and 1 h groups were normally distributed (*p* = 0.261, *p* = 0.068, respectively). However, no further statistical analyses were performed on the 1 and 2 h groups because there were only two and one animal respectively in each of these groups with MCAo + AChAo. There were significant differences in infarct volume between animals with MCAo + AChAo compared to MCAo alone in the pMCAo group (42.7±27.62 mm^3^ vs. 144.5±11.72 mm^3^, *p*<0.0001, Figure 3A). When infarct volume data from the 1 h, 2 h and pMCAo groups were pooled into MCAo and MCAo + AChAo groups, there was a significant difference in infarct volume (24.6±23.38 mm^3^ vs. 129.6±41.58 mm^3^, *p*<0.0001).

AChAo patency and MCAo duration were significantly correlated with infarct volume (r = 0.85, *p*<0.0001; r = 0.58, *p* = 0.0013, respectively). There was no significant correlation between weight and infarct volume (r = –0.22, *p*>0.10). AChAo patency and MCAo duration were included in the subsequent multiple linear regression model, which had an adjusted r^2^ value of 0.831. AChA patency and occlusion duration were both found to be significant independent predictors of 24 h infarct volume with β = 0.732 and 0.367 respectively (both *p*<0.001).

Interestingly, the increase of infarct volume in the MCAo + AChAo group was not restricted to the territory of the AChA, which is entirely subcortical, but was also seen in cortical regions (Figure 2). Quantification of cortical and sub-cortical volumes of infarction in the pMCAo group revealed significantly larger subcortical infarct volumes (59.3±9.11 mm^3^ vs. 28.5±15.93 mm^3^, *p* = 0.001), and even more dramatic differences in cortical infarct volumes in the MCAo + AChAo animals compared to those with MCAo alone (85.2±9.50 mm^3^ vs. 14.2±16.98 mm^3^, *p*<0.00001; Figure 3B & C).

## Discussion

In this study, our goal was to determine the importance of inadvertent AChAo to the major problem of infarct volume variability in the intraluminal thread occlusion model. We showed that it had a major effect. Presence of anterior choroidal artery occlusion on CTP-derived CBV maps was a strong independent predictor of final infarct volume. The relationship between AChAo and infarct volume was present with either permanent or temporary occlusion. Although the known relationship between MCAo duration and infarct volume was also observed, within each occlusion duration group animals with AChAo had larger infarcts than those without. The use of CTP to define anterior choroidal artery occlusion has provided the likely explanation for the dichotomous distribution of infarct volumes after intraluminal thread occlusion of the MCA in rats [11], [13], [14]_ENREF_11.

Occluding filament design, variability of vascular anatomy, and occluder tip positioning are likely causes for variability of AChAo. In the current study, variable occlusion of the AChA during MCAo occurred using occluding tips with consistent silicone tip length and diameter (3 mm×0.35 mm, respectively). Previous studies have shown that occluder tips with larger *diameters* produce larger infarct volumes [8], [15], [16]. Variability of infarct volumes with different tip *lengths* has also been investigated [3]. Combined MCAo and AChAo was reported to occur with occluder tip lengths >3.3 mm, but not with shorter tip lengths [3]. However, studies have also shown that the distance between the AChA and MCA is variable within and across rat strains [3], [17]. Another important variable may be the final positioning of the occluding thread. Ma *et al* (2006) demonstrated that the final position of silicone-tipped occluders (with constant tip lengths), varied within and between six different rat strains, [18].

An important question arising from this research is why AChA occlusion is such an important determinant of infarct volume? Infarction within the arterial territory of the AChA itself does not appear to be a sufficient explanation for this phenomenon. In the rat and human, the anterior choroidal artery typically supplies a small proportion of the sub-cortex and choroid plexus [1], [2]. Although sub-cortical infarction was significantly greater with occlusion of the MCA and AChA, this does not explain why cortical infarction was also significantly larger than MCA occlusion alone. The likely explanation is that occlusion of the AChA prevents collateral flow to the MCA territory via the known MCA-AChA collateral anastomotic vessels [1].

In summary, the intermittent occurrence of inadvertent AChA occlusion during filament occlusion of the MCA may be a major contributor to infarct volume variability. Techniques such as CTP can be used to determine the presence or absence of AChA occlusion. We hope that this finding helps to alert researchers to this important predictor of infarct volume and hence potential cause of model variability. This may enable other laboratories to tailor their model to reduce outcome variability, improve statistical power and reduce sample sizes in preclinical stroke research.
